# Late Effects of Breast Cancer Treatment and Outcome after Corrective Interventions

**DOI:** 10.31557/APJCP.2019.20.9.2673

**Published:** 2019

**Authors:** Unni S Pillai, Smita Kayal, Sunu Cyriac, Yadav Nisha, Kadambari Dharanipragada, Sadish Kumar Kamalanathan, Dhanapathi Halanaik, Navin Kumar, Ponraj Madasamy, Dhanraju Krishnappa Muniswamy, Biswajit Dubashi

**Affiliations:** 1 *Department of Medical Oncology,*; 2 *Department of Surgery,*; 3 *Department of Endocrinology, *; 4 *Department of Nuclear Medicine,*; 5 *Department of Physiology, Jawaharlal Institute of Post Graduate Medical Education & Research (JIPMER), Puducherry, India.*

**Keywords:** Late effects, breast cancer, quality of life

## Abstract

**Purpose:**

To study the late toxicities of treatment and its impact on Breast cancer survivors among Indian patients.

**Materials and Methods:**

Our study recruited 152 curatively treated non metastatic carcinoma breast patients. The baseline demographic details, disease related and treatment related information were collected. The late effects included breast cancer related lymphedema, shoulder dysfunction, treatment induced bone loss, hypothyroidism, cardiac dysfunction, and chemotherapy induced cognitive dysfunction and Quality of life.

**Results:**

The median age was 47 years (range 27 -72 years). The cumulative frequency of BCRL and shoulder dysfunction was 31.57% and 34.86% respectively. The improvement in BCRL with corrective intervention was not statistically significant. The BCRL was significantly associated with shoulder dysfunction. The frequency of loss of bone mineral density was 38.15%. There was statistically significant improvement in bone mineral density with interventions. The cumulative rate of hypothyroidism and cardiac dysfunction was 14.47 % and 2.17% respectively which improved after corrective therapy. We did not find any delayed cognitive dysfunction. There was improvement in global health, physical function, role function, fatigue, Nausea, vomiting, pain scores, insomnia, Loss of appetite, diarrhea and arm symptoms over time with intervention.

**Conclusion:**

Our study has shown that nearly half of the survivors were suffering from at least one of the late effects. The intervention helped in improving the loss of bone mineral density, hypothyroidism, cardiac dysfunction and quality of life in Breast cancer survivors.

## Introduction

Breast cancer (BC) is the commonest non dermatological malignancy among women globally (Parkin et al., 2005; Ferlay et al., 2010; Ferlay et al., 2015). The incidence of BC is increasing in India and it has replaced carcinoma cervix as the most common malignancy among women in most of the cancer registries in India (Das et al., 2014). The current treatment of BC involves surgery with varying combinations of chemotherapy (CT), radiotherapy (RT), anti Her 2 neu treatment and hormonal treatment (HT) depending on the stage of the disease and other predictive parameters. In contrast to the developed nations, we have a huge burden of advanced breast cancers in India, which requires multimodality treatment. The current advances in treatment have improved the outcome of patients diagnosed with BC. This improvement in outcome may be thwarted by the development of potential life threatening long term toxicities.

There is an increasing awareness about the delayed toxicities of cancer treatment and recently it has been extensively studied. The surviving patients are at higher risk of developing premature menopause, lymphedema of the arm, impaired shoulder mobility, loss of bone mineral density, cognitive impairment, cardiac dysfunction and hypothyroidism. The long term follow up has also showed a significant increase in non-cancer morbidity and mortality, which is attributed to the toxicities of treatment. Unfortunately the late toxicities of treatment and its impact on BC survivors were not studied among Indian patients. There is also a need to understand whether the late effects can be corrected and will this lead to an improvement in outcome.

## Materials and Methods

*Participant*

This study was a prospective cohort study. Written Informed Consent was taken from all subjects prior to the inclusion in the study. The nature and importance of the study was explained to the subjects. 

*Inclusion criteria*

- Diagnosed invasive breast cancer patients who had completed treatment except hormonal treatment and on follow up for at least 1 year.

*Exclusion criteria*

- Metastatic disease

- Any local or distant failure

- Any other prior history of malignancy

The base line demographic details, disease related and treatment related information were collected from the case record. The patients were evaluated for late effects of treatment and corrective interventions were administrated to those who were found to have late effects. The late effects were breast cancer related lymphedema (BCRL), shoulder dysfunction, treatment induced bone loss, hypothyroidism, cardiac dysfunction and chemotherapy induced cognitive dysfunction. The QoL of all the patients were collected using EORTC QLQ C30 and BR 23 at the time of study entry and there after 12 months.

*Breast cancer related lymphedema*

The patients were evaluated for BCRL with a measuring tape and the arm circumferences were measured at four fixed points. The points were 5 and 10 cm above and below the lateral epicondyle of the elbow joint. The measurements were done on both the upper limbs and any difference of 2 cm was recorded as lymph edema. The lymph edema was further graded as follows

Grade 1 represent Maximum difference in arm circumference is 2-5cm,

Grade 2 represent Maximum difference in arm circumference is 5.1-8cm,

Grade 3 represent Maximum difference in arm circumference is 8.1- 11cm,

Grade 4 represent Maximum difference in arm circumference is > 11cm.

Those patients who were diagnosed with BCRL were further offered treatment with manual compression drainage and elastic compression crepe bandages. The lymph edema was recorded in each visit and was compared with the base line.

*Shoulder dysfunction*

Shoulder dysfunction was measured using Common toxicity criteria for adverse events (CTCAE) version 4.03 Those patients who were diagnosed with shoulder dysfunction were offered physical medicine treatment with graded exercise and mobilization of the affected joint. 

*Loss of bone mineral density*

Bone mineral density (BMD) was measured using DEXA Scan (Kanis, 1994). The patients who were diagnosed with osteopenia or osteoporosis were offered Calcium , vitamin D3 supplementation (1,000 mg and 400 mcg respectively) and zolendronic acid every 6 month. The DEXA scan was repeated every year to find out the changes from the base line.

*Hypothyroidism*

Patients were evaluated for hypothyroidism with a free T4 and TSH level study at baseline. The thyroid function test was repeated every month. 

*Cardiac dysfunction*

Cardiac dysfunction was assessed with multi gated acquisition scan (MUGA Scan) using Tc 99 labelled RBCs. The test was offered to all the patients at base line. The criteria for diagnosis of cardiac dysfunction were left ventricular ejection fraction less than 50%.

*Chemotherapy induced cognitive dysfunction*

Chemotherapy induced cognitive dysfunction was screened with a mini mental score examination (MMSE) at the OPD. MMSE is used to screen for cognitive dysfunction in patients with dementia and the score ranges from 0 to 30. The definition of cognitive dysfunction in our study was an MMSE score of ≤ 24. 

*Quality of life*

The quality of life of the patient was recorded using EORTC QLQ C 30 and BR 23. The regional validated Tamil version of the questionnaire was printed and given to patients at the time of study entry and after 12 month. If the patient was illiterate, the investigator and medical social worker interviewed the patient and the response was recorded. The scoring was according to the EORTC QLQ scoring manual for individual functional and symptom scale parameters. The higher score for item represents a higher level of function for function item and worst symptom for symptom item.

*Statistical Analysis*

Analyses were done for two-tailed significance at 5% level of significance and p value < 0.05 was considered as significant. All statistical analyses were carried out using SPSS version 16.

## Results

152 patients were enrolled into our study. The median age was 47 years (range 27 -72 years). 71.1% patients were having locally advanced BC and 94.6% had mastectomy. All the patients had undergone complete axillary dissection and 69.1% were also treated with radiotherapy. 53.28% patients were having ER positive disease and 61.84%were treated with adjuvant hormonal treatment. 98.02% patients had chemotherapy for breast cancer and 66.65% patients received a combination chemotherapy containing anthracycline and taxanes are shown in Table 1.

**Table 1:** Baseline Patient Characteristics

Total number of patients (N)	152
Median age	47 yrs (28-72 yrs)
Median time from end of the treatment to study entry	33 months (12-100 months)
Median follow up in the study	18 months (12- 24 months)
Stage of the disease	
Early breast cancer	44 (28.9%)
Locally advanced breast cancer	108 (71.1%)
Hormone receptor	
Positive	91 (59.86%)
Negative	58 (38.15%)
unknown	3 (1.97%)
Her 2 neu*	
1+	85 (55.92)
2+	43 (28.28)
3+	24 (15.78%)
Type of surgery	
MRM	144 (94.6%)
BCS	8 (5.3%)
Radiotherapy	
No radiotherapy	47 (30.9%)
Adjuvant radiotherapy	105 (69.1 %)
Chemotherapy	
With taxane	105 (69.07%)
Without taxane	44 (28.94%)
No chemotherapy	3 (1.9%)
Adjuvant hormonal treatment	
Tamoxifen	47 (30.4%)
Aromatase inhibitor	44 (28.94)%

++ The median time from end of the treatment to study entry was 33 months (range 12-100 months) and median study duration was 18 months (12-24 months).The overall frequency of co-morbidities at the time of breast cancer diagnosis was 20.39 %, which included 13.8% patients with hypertension, 9.86% patients with diabetes and 3% patients with hypothyroidism and 18.42% with diabetes and hypertension. The cumulative incidence of all late effects was 51.31%. The cumulative frequency of late effects was 48.02%, with loss of bone mineral density was 38.15% as the most common late effect. The cumulative frequencies of shoulder dysfunction were 34.86%, BCRL was 31.57%, hypothyroidism was 14.47% and cardiac dysfunction was 2.17%. We did not find any cognitive dysfunction.

**Table 2:** Association between Different Baseline Parameters and Development of Shoulder Dysfunction

Parameter (N=152)	Without shoulder dysfunction N=99 (65.13%)	With Shoulder dysfunction N=53 (34.68%)	p value (univariate analysis)	p value (multivariate analysis)
Stage			0.38	
Early (44)	33 (75%)	13 (25%)		-
Locally advanced (108)	68 (62.96%)	40 (37.04%)		
Adjuvant radiotherapy			0.047*	
Received(105)	63 (60%)	42 (40%)		-
Not received (47)	36 (76.95%)	11 (23.40%)		
Surgery			0.714	
MRM (144)	93 (64.58%)	51 (35.41%)		_
BCS (8)	6 (75%)	2 (25%)		
BCRL			<0.001**	<0.001**
Present (48)	13 (27.08%)	35 (79.16%)		
Absent (104)	86 (80.37%)	18 (17.30%)		

**P < 0.001 , * P < 0.05 were considered statistical significant; ++ The frequency of shoulder dysfunction was significantly associated with BCRL in both univariate and multivariate analyses but not significantly associated with any of the other baseline parameters as shown in table 2..

**Table 3:** Comparison of Bone Mineral Density among Postmenopausal Women at Baseline and after 12 Months

	DEXA scan 12 months			Significance
DEXA scan at base line (N=105)	Normal	Osteopenia	Osteoporosis	(Mc Nemar Bowker chi square test)
Normal (57)	57	0	0	0.007*
Osteopenia (29)	5	24	0	
Osteoporosis (19)	0	5	14	
Total	62	29	14	

* P < 0.05 was considered statistical significant; ++The cumulative frequency of all the grades of decreased BMD was 38.15 %.This includes 25.65% osteopenia and 12.5% osteoporosis. There was no pathological fracture. All the patients were asymptomatic despite decreased bone mineral density. After intervention with Zolendronic acid there was significant improvement in the BMD.

**Table 4:** Factors Associated with the Development of Decreased Bone Mineral Density

Parameter	Normal bone density (N=94)	Decreased bone density (N=58)	Odds ratio (95% CI)	P value (univariate)	P value (multivariate)
Menopausal status					0.007*
Postmenopausal (105)	57	48	3.115	0.004*	
Premenopausal (47)	37	10	(1.404-6.914)		
Adjuvant hormonal treatment					0.009*
Received (94)	50	44	2.765	0.005*	
Not received (58)	44	14	(1.339-5.711)		
Chemotherapy					
Taxane containing (105)	64	41	1.13	0.74	-
Without taxane (47)	30	17	(0.554-2.305)		
Hormonal treatment					-
Letrezole (49)	24	25	1.425	0.392	
Tamoxifen (45)	26	19	(0.631-3.21)		

* P < 0.05 was considered statistical significant; ++, There was significant association of decreased BMD and adjuvant hormonal treatment. Postmenopausal status was also significantly associated with the loss of bone mineral density. There was no significant difference in terms of BMD between Tamoxifen and Letrozole group.

**Table 5:** Comparison of Quality of Life by EORTC BR 23 at Baseline and Over 12 Months

(N=152)	Base line	12 months	p- value
Body image	76.8± 10.4	76.9±12.5	0.87
Sexual functional	39.2±39.3	36.5± 34.1	0.3
Sexual enjoyment	31.7±33.5	30.5±34.8	0.45
Future perspective	66.7±24.3	65.2±26.4	0.11
Systemic therapy side effects	14.1±9.9	11.2±9.9	0.01*
Breast symptoms	28.1±15.3	27.3±17.3	0.81
Arm symptoms	30.1±21.1	25.1±20.3	0.01*
Upset with hair loss	1.7±7.3	3.4±11.5	0.01*

* P value < 0.05 was considered as statically significant; ++ There was improvement in EORTC QLQ BR 23 score for systemic therapy side effects and arm symptoms at 12 months compared to base line.

*BCRL*

The cumulative frequency of all the grades of BCRL was 48 (31.6%). The frequency of BCRL was 31.6%, 31.6%, 29.1%,33.0% and 32.4% respectively at 0, 6, 12, 18 and 24 months. There was no significant change in the frequency of BCRL with time.

Most of the patients with lymphedema had grade 1 lymph edema (26.3%) with a cumulative frequency of 5.3% for grade 2 to grade 4 lymphedema. There was no improvement in the frequency of BCRL with our intervention. There was no statistically significant difference in the mean arm circumference at 6 months or at 12 months compared to baseline. The frequency of BCRL was not significantly associated with any of the baseline parameters in our study. 

*Shoulder dysfunction*

Patients with shoulder dysfunction had grade 1 late effect (27.7%) with cumulative frequency of 7.2 % for grade 3 shoulder dysfunction. There was no improvement in the frequency of shoulder dysfunction with our intervention at 6 months (p=0.580) or at 12 months (p=0.126). The mean degree of restriction of shoulder flexion was 32^o^C and that of abduction was 41^o^C, for patients with shoulder dysfunction at baseline. There was no statistically significant difference in the mean arm circumferences at 6 months or at 12 months compared to the baseline represented in the Table 2.

*Loss of Bone Mineral Density*

The cumulative frequency of all the grades of decreased BMD was 38.15 %.This includes 25.65% osteopenia and 12.5% osteoporosis are shown in Table 3. There was no pathological fracture. We also analyzed the association of baseline disease and treatment related parameters to the development of decreased BMD (Table 4).

*Hypothyroidism*

The prevalence of symptomatic thyroid disease on treatment at the time of BC diagnosis was 4%. After a median follow up of 33 months of curative treatment, the frequency of subclinical hypothyroidism among BC survivors was 9.2% and overt hypothyroidism was 5.26%, with overall prevalence of hypothyroidism of 14.47%. All these patients were having asymptomatic hypothyroidism and treatment with thyroxin resulted in prompt reversal of hypothyroidism within 6 months of follow up. The type of chemotherapy and surgical treatment of breast cancer was not associated with an increase in hypothyroidism. 

*Quality of life*

The quality of life was assessed with EORTC QLQ C 30 and BR 23 questionnaire. The score for financial difficulty was higher than the large population based reference value in our study, reflecting the socio economical parameters of our patient population.

The scores for the other symptom parameters were comparable to the population based values. We compared the quality of life at base line and 12 months to find out whether our interventions to alleviate the late effects resulted in improvement in quality of life are shown in table 5. There was significant improvement in EORTC QLQ C 30 scores except Emotional function, Social function, dyspnea and constipation at 12 months compared to the base line.

*Cognitive dysfunction*

The MMSE was used to screen the cognitive dysfunction. The mean MMSE scores were 28.71, 28.38, 29.32, 28.4, and 28.56 at study entry, 6, 12, 18 and 24 months respectively. The MMSE scores of all the patients were above 24, cut off value for sending the patients for a battery of neurocognitive tests. 

*Late cardiac dysfunction*

The cardiac dysfunction was assessed by MUGA scan and the mean LVEF was 59.11% (±4.381). We identified two patients with ejection fraction < 50%. Both patients had history of long standing hypertension and were on irregular treatment. They were started on ACE inhibitors and β2 blockers after cardiology evaluation. The follow up scans were done only for these two patients and there were improvement in ejection fraction > 50%. 

## Discussion

In our study nearly half of the patients were having at least one late effect. The most common late effect was loss of BMD followed by shoulder dysfunction, BCRL, hypothyroidism and cardiac dysfunction in decreasing frequencies. The patients were having excellent quality of life. In respect to BCRL, there are only a few studies addressing the issue of late lymphedema and morbidities after the curative treatment of breast cancer (Stout et al., 2012; Scaffidi et al., 2012 ). 

Lymphedema is a significant problem in curatively treated BC patients and is associated with a lower quality of life (Freitas-Silva et al., 2010; Nesvold et al., 2010). The incidence of BCRL in other studies ranges from 15-35% and varies with the definition of lymph edema and the method used to evaluate BCRL (Ancukiewicz et al., 2011). Physiotherapy and exercise treatment were not significantly associated with an improvement in lymphedema in our study. These results are in concordance with the available evidence that the established lymphedema are difficult to treat ( Nesvold et al., 2008; Nesvold et al., 2010; De Groef ., et al 2015). 

Shoulder dysfunction is a major morbidity and our results confirm this established findings (Nesvold et al., 2008; Sagen et al., 2009). It is estimated that 90% of late shoulder morbidity developed during the first 3.9 years. The median observation time of 33 months was sufficient for including majority of those patients who might develop long-term impairment in arm/shoulder function. The incidence of grade 3 shoulder dysfunction was 7.2%. There was no improvement in shoulder function with physical therapy and this suggests that the shoulder morbidities are better prevented than treated. Our study confirms that the interventions to tackle established shoulder dysfunction are less effective. A limited axillary surgery with sentinel lymph node biopsy and locally less aggressive surgeries results in improved shoulder function. Early shoulder rehabilitation after BC surgery has also resulted in improved shoulder function among BC survivors (Scaffidi et al., 2012). 

The cumulative frequency of loss of BMD in our study was 38.15%. This is comparable to the frequencies of bone loss reported in other studies. The incidence of bone loss in the control arm of the alliance trial was 41.2% (Brufsky et al., 2012). All the patients were asymptomatic and there were no pathological fractures. One difference in our approach compared to randomized trial is that we have offered treatment only to those patients who were found to have decreased BMD. The dosing of zolendronic acid was different in various studies and we used once in every 6 months schedule.

The variable significantly associated with the decreased BMD in our study was adjuvant hormonal treatment and postmenopausal status. Most of the patients were received aromatase inhibitor at the time of study entry. Other variables which were found to be associated with decreased BMD in other studies were not found to have a significant impact on the development of loss of BMD in our study, which may be due to the small sample size and relatively short period of follow up. 

In our study the cumulative frequency of hypothyroidism was 14.86% and the incidence may rise with long term follow up. All patients had asymptomatic hypothyroidism, with 15.26% having overt biochemical hypothyroidism and 9.2% subclinical hypothyroidism. The incidence in other studies are in the range of 20% at median follow up of 7 years (Kumpulainen et al., 2000). The probability of hypothyroidism may increase with increasing age and studies have shown that the risk is greater among females (Smith et al., 2008). The relatively small volumes with high radiation exposure may also increase the risk of hypothyroidism in these patients (Johansen et al., 2011). Whether chemotherapy increases the risk of hypothyroidism is unclear. Kumar et al., (2004) suggested that cytotoxic chemotherapy may influence thyroid function in cancer patients leading to progressive symptomatic worsening.

The incidence of CD in our present study was just 2.17% and all these patients were having asymptomatic CD except that they had a history of hypertension. The most important aspect was that all the patients with CD were promptly reversed by treatment with ARBs and β blockers. None of the patients developed symptomatic HF or had cardiac related mortality. But the results were not in concordance with the results from large series published from the west. 

An important aspect in our study is that the screening with MMSE failed to demonstrate any late cognitive dysfunction among BC survivors. Many studies have demonstrated that the cognitive deficits in BC survivors are subtle and involving complex neural network and affecting multiple domains (Folsteine et al., 1975). This may lead to significantly worse abstract thinking, memory and analytical function. It has also been demonstrated that the late cognitive effects are maximum at the extremes of life (Vodermaier, 2009 ; Champion et al., 2014; Piccirillo et al., 2015).

We examined QOL among surviving BC patients and confirmed findings in other studies that the BC survivors has high quality of life parameters and are relatively asymptomatic on long term follow up.

In our study the mean global health score, Physical function score , Role function score, Emotional function score ,Cognitive function score and Social function score is comparable to the large population based reference value (Avis et al., 2005). But these values are better than the values reported for patients on breast cancer treatment (Saleha et al., 2012). The patients in our study are relatively symptomatic for all the parameters except dyspnea and constipation. The score for financial difficulty was higher than the large population based reference value reflecting the socio economical parameters of our patient population. The scores for the other symptom parameters were comparable to the population based value. All the symptom scores were less compared to the reported values for the patients who were on treatment, indicating that the tumor and treatment related symptoms recover to a greater extent over time.

In our study the EORTC BR 23 function scores for body image and future perspective are comparable to that reported in the literature but the score for sexual functioning and sexual enjoyment are less than that reported in the literature. This may be due to the fact that the median age in our population was 47yrs and majority of the patients had menopause (Ghezzi et al., 1994). Regarding the size of the symptoms of EORTC BR23, there was a significant improvement in symptoms in the arms and breasts during follow-up and worsening of upset on hair loss during our study period. These findings suggest that the interventions might have helped to improve the QoL of these patients and reflecting the late BCRL and shoulder dysfunction.

In conclusion, 152 non metastatic BC patients on follow up after curative treatment has shown that nearly half of the survivors are suffering from at least one of the late effects. The intervention helps in improving loss of bone mineral density, hypothyroidism, cardiac dysfunction and quality of life of BC survivors. We did not find any significant improvement in BCRL and shoulder dysfunction with our given interventions and it may require longer duration of interventions and follow up for improvement of these conditions. 
